# 2371. A study to describe the pattern of cutaneous adverse effects of COVID-19 vaccines (Covishield and Covaxin )

**DOI:** 10.1093/ofid/ofad500.1992

**Published:** 2023-11-27

**Authors:** Debdeep Mitra, Neerja Saraswat

**Affiliations:** Command Hospital Air Force Bangalore, Bangalore, Karnataka, India; Command Hospital Northern Command, Udhampur, Jammu and Kashmir, India

## Abstract

**Background:**

Vigorous administration of Covid-19 vaccines to tackle the ongoing pandemic has led to increasing research on adverse effects including both systemic and cutaneous.

A prospective observational study to delineate cutaneous adverse effects of two vaccines namely Covishield and Covaxin administered in two doses in northern India.

**Methods:**

The study was conducted in a tertiary hospital in northern India wherein patients were asked to report voluntarily any cutaneous adverse effects after covid-19 vaccination to the dermatology department. The data was collected using excel sheets and later analysed taking into consideration the age, vaccine types, and duration of onset of adverse effects.

**Results:**

Out of the 19672 vaccination jabs, 296 (1.5%) developed cutaneous adverse effects out of which the incidence was higher in Covishield vaccinees compared to Covaxin vaccinee group. The incidence of side effects was more with the first dose of either vaccine compared to the second dose. All the side effects were benign and were managed symptomatically or were self-limiting.

**Conclusion:**

Rampant administration of vaccines along with widespread advertisement of vaccine-induced side effects via social media has created apprehension in the general population. This warrants studies improving awareness about the most vital preventive measure available to halt and eventually end the Covid-19 pandemic.

**Disclosures:**

**All Authors**: No reported disclosures

